# Supplementary material for: Discovery and application of insertion-deletion (INDEL) polymorphisms for QTL mapping of early life-history traits in Atlantic salmon
Source: BMC Genomics. 2010 Mar 8;11:156. doi: 10.1186/1471-2164-11-156 (PMC2838853; doi:10.1186/1471-2164-11-156)
Supplement: Additional file 2 — Information on developed 76 locus single-run INDEL panel in Atlantic salmon. Information on fluorescence labeling, primer concentrations, PCR pooling and links to alignments, INDEL motifs and GENESCAN (Burge and Karlin 1997) predictions of genes/exons are available in html format. [file 1471-2164-11-156-S2.ZIP › Additionalfile2/snpsummary4319.html]

```
Cluster 1000 Contig 2

prev  Summary    Contig List  next
```

Size of Consensus sequence = 1839

Number of sequences = 33

Minimum redundancy = 6

Key

A gi|45310018|gb|CK880387.1|CK880387 SGP140688 Atlantic salmon Head kidney cDNA library Salmo salar cDNA clone FN5-0226 5', mRNA sequence  
B gi|117845249|gb|EG917945.1|EG917945 EST\_ssal\_evf\_57048 ssalevf mixed\_tissue Salmo salar cDNA Salmo salar cDNA clone ssal\_evf\_576\_194\_rev 5', mRNA sequence  
C gi|117476757|gb|EG808976.1|EG808976 EST\_ssal\_evd\_29399 ssalevd thymus Salmo salar cDNA Salmo salar cDNA clone ssal\_evd\_538\_211\_rev 5', mRNA sequence  
D gi|117845248|gb|EG917944.1|EG917944 EST\_ssal\_evf\_57047 ssalevf mixed\_tissue Salmo salar cDNA Salmo salar cDNA clone ssal\_evf\_576\_194\_fwd 3', mRNA sequence  
E gi|117830729|gb|EG903425.1|EG903425 EST\_ssal\_evf\_41984 ssalevf mixed\_tissue Salmo salar cDNA Salmo salar cDNA clone ssal\_evf\_556\_026\_rev 5', mRNA sequence  
F gi|89869381|gb|DY725504.1|DY725504 EST\_ssal\_rgb2\_81243 ssalrgb2 mixed\_tissue Salmo salar cDNA Salmo salar cDNA clone ssal\_rgb2\_633\_170\_fwd 3', mRNA sequence  
G gi|85020303|gb|DW548959.1|DW548959 EST\_ssal\_rgb2\_13378 rgb2 Salmo salar cDNA clone ssal\_rgb2\_522\_139\_fwd 3', mRNA sequence  
H gi|117835518|gb|EG908214.1|EG908214 EST\_ssal\_evf\_10550 ssalevf mixed\_tissue Salmo salar cDNA Salmo salar cDNA clone ssal\_evf\_512\_190\_rev 5', mRNA sequence  
I gi|117537743|gb|EG869188.1|EG869188 EST\_ssal\_eve\_30461 ssaleve thyroid Salmo salar cDNA Salmo salar cDNA clone ssal\_eve\_541\_142\_rev 5', mRNA sequence  
J gi|85030457|gb|DW559113.1|DW559113 EST\_ssal\_rgb2\_23532 rgb2 Salmo salar cDNA clone ssal\_rgb2\_538\_082\_rev 5', mRNA sequence  
K gi|45310918|gb|CK881287.1|CK881287 SGP141623 Atlantic salmon Head kidney cDNA library Salmo salar cDNA clone FN4-0693 5', mRNA sequence  
L gi|29324222|gb|CB512996.1|CB512996 ssalrgb544259\_rev mixed\_tissue Salmo salar cDNA, mRNA sequence  
M gi|45307078|gb|CK877447.1|CK877447 SGP138761 Atlantic salmon Gills cDNA library Salmo salar cDNA clone G6-0436 5', mRNA sequence  
N gi|89838044|gb|DY696962.1|DY696962 EST\_ssal\_rgb2\_52701 ssalrgb2 mixed\_tissue Salmo salar cDNA Salmo salar cDNA clone ssal\_rgb2\_585\_097\_rev 5', mRNA sequence  
O gi|117848511|gb|EG921207.1|EG921207 EST\_ssal\_evf\_443 ssalevf mixed\_tissue Salmo salar cDNA Salmo salar cDNA clone ssal\_evf\_003\_046\_fwd 3', mRNA sequence  
P gi|89847719|gb|DY703842.1|DY703842 EST\_ssal\_rgb2\_59581 ssalrgb2 mixed\_tissue Salmo salar cDNA Salmo salar cDNA clone ssal\_rgb2\_596\_002\_rev 5', mRNA sequence  
Q gi|117487840|gb|EG820057.1|EG820057 EST\_ssal\_evd\_22031 ssalevd thymus Salmo salar cDNA Salmo salar cDNA clone ssal\_evd\_528\_187\_fwd 3', mRNA sequence  
R gi|117830730|gb|EG903426.1|EG903426 EST\_ssal\_evf\_41985 ssalevf mixed\_tissue Salmo salar cDNA Salmo salar cDNA clone ssal\_evf\_556\_026\_fwd 3', mRNA sequence  
S gi|117835519|gb|EG908215.1|EG908215 EST\_ssal\_evf\_10551 ssalevf mixed\_tissue Salmo salar cDNA Salmo salar cDNA clone ssal\_evf\_512\_190\_fwd 3', mRNA sequence  
T gi|117506206|gb|EG837965.1|EG837965 EST\_ssal\_eve\_9365 ssaleve thyroid Salmo salar cDNA Salmo salar cDNA clone ssal\_eve\_512\_256\_fwd 3', mRNA sequence  
U gi|85042482|gb|DW570660.1|DW570660 EST\_ssal\_rgb2\_35079 rgb2 Salmo salar cDNA clone ssal\_rgb2\_556\_290\_rev 5', mRNA sequence  
V gi|57121366|gb|CX352807.1|CX352807 ssalrgb527175\_rev\_0 mixed\_tissue Salmo salar cDNA, mRNA sequence  
W gi|117537744|gb|EG869189.1|EG869189 EST\_ssal\_eve\_30462 ssaleve thyroid Salmo salar cDNA Salmo salar cDNA clone ssal\_eve\_541\_142\_fwd 3', mRNA sequence  
X gi|12621773|gb|BF228584.1|BF228584 EST00295 Atlantic salmon Lambda Zap Express brain cDNA library Salmo salar cDNA clone BNS-05 5', mRNA sequence  
Y gi|85020302|gb|DW548958.1|DW548958 EST\_ssal\_rgb2\_13377 rgb2 Salmo salar cDNA clone ssal\_rgb2\_522\_139\_rev 5', mRNA sequence  
Z gi|15846451|gb|BG936623.1|BG936623 SS1-1006 Atlantic Salmon spleen Salmo salar cDNA clone SS1-1006 similar to SKP1, mRNA sequence  
a gi|89869382|gb|DY725505.1|DY725505 EST\_ssal\_rgb2\_81244 ssalrgb2 mixed\_tissue Salmo salar cDNA Salmo salar cDNA clone ssal\_rgb2\_633\_170\_rev 5', mRNA sequence  
b gi|29324713|gb|CB513487.1|CB513487 ssalrgb527049\_rev mixed\_tissue Salmo salar cDNA, mRNA sequence  
c gi|85018605|gb|DW547261.1|DW547261 EST\_ssal\_rgb2\_11680 rgb2 Salmo salar cDNA clone ssal\_rgb2\_519\_286\_rev 5', mRNA sequence  
d gi|89879255|gb|DY735378.1|DY735378 EST\_ssal\_rgb2\_91117 ssalrgb2 mixed\_tissue Salmo salar cDNA Salmo salar cDNA clone ssal\_rgb2\_649\_071\_rev 5', mRNA sequence  
e gi|117515042|gb|EG846801.1|EG846801 EST\_ssal\_eve\_713 ssaleve thyroid Salmo salar cDNA Salmo salar cDNA clone ssal\_eve\_006\_009\_fwd 3', mRNA sequence  
f gi|117475866|gb|EG808085.1|EG808085 EST\_ssal\_evd\_28597 ssalevd thymus Salmo salar cDNA Salmo salar cDNA clone ssal\_evd\_537\_169\_fwd 3', mRNA sequence  
g gi|117832698|gb|EG905394.1|EG905394 EST\_ssal\_evf\_8012 ssalevf mixed\_tissue Salmo salar cDNA Salmo salar cDNA clone ssal\_evf\_509\_022\_fwd 3', mRNA sequence

3 SNPs detected

A B C D E F G H I J K L M N O P Q R S T U V W X Y Z a b c d e f g  cosegregation weighted

1317 . . . . . . . . . . . . . . - - C C - C - - C C . C - - - C - - -   3/3 54.55
1318 . . . . . . . . . . . . . . - - T T - T - - T T . T - - - T - - -   3/3 54.55
1319 . . . . . . . . . . . . . . - - G G - G - - G G . G - - - G - - -   3/3 54.55
